# Inequality in provider continuity for children by Australian general practitioners

**DOI:** 10.1186/1471-2296-12-106

**Published:** 2011-09-30

**Authors:** Marjan Kljakovic, Karen Ciszek, Graham Reynolds, Samuel Colman

**Affiliations:** 1 Academic Unit of General Practice and Community Health in the School of General Practice, Rural and Indigenous Health, at the Australian National University Medical School, PO Box 11 Woden, ACT 2606, Australia; 2 Department of Paediatrics at the Canberra Hospital, Australian National University Medical School, PO Box 11 Woden, ACT 2606, Australia; 3 Biostatistics, Covance Pty Ltd Level 3, 4 Research Park Drive, North Ryde, NSW 2113, Australia

## Abstract

**Background:**

There is little published on provider continuity in Australian general practice and none on its effect on inequality of care for children.

**Method:**

Questionnaire administered to parents of the ACT Kindergarten Health Screen asking the name of their child's usual GP and practice address between 2001 and 2008.

**Results:**

Parents of 30,789 children named 433 GPs and 141 practices. In each year, an average of 77% of parents could name both the GP and the practice, an average of 11% of parents could name only the practice, and an average of 12% of parents could name neither. In each year, 25% of parents could not name a usual GP for children of Aboriginal or Torres Straight Islander descent, or children born outside of Australia, compared to 10% of all other children (p = < 0.0001). The frequency of GPs displaying continuity of care varied over time with 19% of GPs being present in the ACT in only one year and 39% of GPs being present in every year over the eight years of study. GPs displayed two different forms of transience either by working in more than one practice in each year (5% of GPs), or by not being present in the ACT region from one year to the next (15% of GPs). Fewer parents nominated transient GPs as their child's GP compared to choosing GPs who displayed continuity (p < 0.001).

**Conclusions:**

Many GPs (39%) were reported to provide continuity of care for in the ACT region and some GPs (20%) displayed transient care. Indigenous children or children born outside of Australia had less equity of access to a nominated GP than all other children. Such inequity might disappear if voluntary registration of children was adopted in Australian general practice.

## Background

The story of the GP who remained in one location managing the many health problems people present over time is a story unique to general practice. The theme of continuity of care was first described in Holland [1] and more recently weaves through stories of Australian general practices published on the RACGP website [2]. A recent systematic review defined continuity of care as a three dimensional description of health care which included informational, longitudinal, and personal continuity of care [3]. Each of these dimensions have been adopted in the 2010 RACGP standards for general practice as an achievable standard in Australia [4], but do not comment on how provider continuity might be achieved by GPs. In Australia, there is no formal process where patients voluntarily register with individual GPs or their practices and thereby create administrative registries. There are a number of indirect methods of counting GPs used by DOHA from an analysis of Medicare data producing paradoxical results [5,6]. For example, the full-time equivalent method of counting individual GPs produces a decrease in numbers of GPs between 2004 and 2008 in the ACT, but the headcount method produces a small increase. None of these methods result in a published account of individual GPs or individual general practices over time. Our study aims to address this gap by analysing systematically gathered names of GPs and their practice addresses. Our first null hypothesis was that similar proportions of GPs display continuity and transient care over time.

A principle policy goal of Medicare is to provide equal care for equal need to all Australian people, for all ages. Nevertheless, previous research has shown that Medicare does not guarantee equity of health care for health related outcomes [7]. Our second study aim was to describe the equity of access to GP care for children in the ACT. Our second null hypothesis was there are no disparities in equity of access to GPs for Indigenous children (who are of Aboriginal or Torres Strait Islander descent), or overseas born, compared to all other children.

## Methods

Each year, all 4 to 6 year old primary school-entry children take part in the ACT Kindergarten Health Screen for a population-based school health assessment, which includes a survey completed by parents (delivery and structure described elsewhere [8]). Since 1998, this survey has comprised a general health questionnaire that includes questions asking the name of the child's GP and the address of the general practice. Response rates of 85% to 89% to the questionnaire are achieved each year. The distribution of all the 105 private and government funded kindergartens match the spread of general practices across the ACT region increasing the likelihood that most GPs and general practices are named each year.

### Defining the naming of GPs

1. '*Naming a GP' *was defined when parents responded yes to the question "Does your child have a usual medical practitioner?" and provided the name of the GP and a practice address.

2. Parents who could not name a GP were defined as '*Not Naming a GP'*.

3. A '*New GP' *was defined as a GP who was not named in any of the previous years.

4. A '*GP left the area' *was defined as a GP who was not named in a current year and never again.

5. A '*GP displayed transient care' *was defined as a GP who was not named in a current year but was named in subsequent years.

Data were collected from parents in each year from 2001 until 2008 inclusive. The data entered into the screening database are cleaned and checked. Each year, a list of names of new GPs and any new practice address (including phone number) is produced by the local General Practice Liaison Unit in the Canberra Hospital. This list is checked against ACT's telephone book for accuracy. Practices were deemed to match against the list if the phone number was the same or the name and address was the same each year. The matching was also visually assessed to correct any mismatching practices. GPs were deemed to match if the full name was the same or last name and first initial were the same. Or the first three characters of the last name and first name were the same. Or the first three characters of the last name and practice were the same when the first name was missing. The matching was also visually assessed to correct any mismatching GPs. The accuracy of the matching was critical to the results. The matching of practices was reliable due to the use of the phone number for matching. The GP matching was dependent on text fields and therefore the level of confidence for accurate matching was reduced.

In 2008, six general practices (named Index General Practices) from the ACT region agreed to measure the concordance between parents naming a GP and practice in the Kindergarten screen and whether the child's name had been recorded in the named practice records.

We undertook descriptive statistics of continuity for the named GPs and their practices between 2001 and 2008. Statistical comparisons were made using chi-squared tests and T-tests for categorical and continuous outcomes respectively. All analysis was undertaken using SAS version 9.1.3.

Ethical approval was obtained from the Australian Nation University Human Ethics Committee and the ACT Health Human Research Ethics Committee.

## Results

Parents of 30,789 children responded to the questionnaire between 2001 and 2008. The mean age of the children was 5.7 years, 50.5% were male, 1.8% were Indigenous children, and 6.3% were children were born outside of Australia. There was an average of 31.3 (95% CI 29.0 to 33.7) children in each general practice named by parents, with a range of 1 to 283 children per practice.

Significantly more parents of children born outside of Australia could not name a usual GP for their child compared to parents of all other Canberra children (27% versus 10% Chi squared = 514.9, df = 1, p = < 0.001). Table 1 shows that this result was consistent in each year over the eight years of study. The proportions varied from one year to the next for parents of Indigenous children. However overall, significantly more parents of Indigenous children could not name a usual GP for their child compared to all other children in Canberra when the results of eight years of study were combined. (17% versus 11%, Chi squared = 19.7, df = 1, p = < 0.001). When the parents of Indigenous children and the parents of children born outside of Australia were combined, significantly more parents of the combined groups could not name a usual GP for their child compared to all other Canberra children (25% versus 10%, Chi squared = 498.9, df = 1, p = < 0.001).

**Table 1:** The percent of parents of Indigenous children, parents of children born outside Australia, and parents of both groups compared to the percent of parents of all other children in the ACT who did not name a usual GP for their child between 2001 and 2008

Year	Parents of Indigenous children			Parents of children born outside Australia			Both groups of parents of Indigenous children or children born outside Australia		
	Yes	No#	p-value*	Yes	No#	p-value*	Yes	No#	p-value*
2001	19%	12%	0.0983	28%	10%	< .0001	26%	10%	< .0001
2002	20%	10%	0.0116	27%	9%	< .0001	26%	9%	< .0001
2003	13%	11%	0.6895	28%	10%	< .0001	24%	10%	< .0001
2004	13%	10%	0.2693	23%	9%	< .0001	21%	9%	< .0001
2005	19%	10%	0.0051	25%	9%	< .0001	24%	9%	< .0001
2006	16%	10%	0.1536	29%	9%	< .0001	27%	9%	< .0001
2007	17%	11%	0.1064	25%	10%	< .0001	24%	9%	< .0001
2008	20%	14%	0.1882	29%	13%	< .0001	27%	13%	< .0001
Total	17%	11%	< .0001	27%	10%	< .0001	25%	10%	< .0001

*Chi squared test.

# Parents of all other children in the ACT

Parents named 433 individual GPs and 141 practice addresses between 2001 and 2008. An average of 77% (95% CI 76.5-77.5) of parents could name both the GP and the practice, an average of 11% (95% CI 10.6-11.3) of parents could name only the practice address, and an average of 12% (95% CI 11.8-12.5) of parents could not name either the child's GP, nor the child's general practice address.

Parents named an average of 2.68 (95% CI 2.55 to 2.81) GPs per practice per year. There was a 15% decline in the number of practices named by parents from 118 in 2001 to 100 in 2008. There was a 13% point decline in solo practitioners named by parents from 42% (n = 49) in 2001 to 29% (n = 29) in 2008. There was a 4% point rise in large general practices (defined as six or more GPs per practice) named by parents from 9% (n = 11) in 2001 to 13% (n = 13) in 2008.

DOHA, AIHW, and Medicare use different methods to measure the number of GPs in the ACT region as listed in Table 2. Our method of counting GPs found 130 fewer GPs each year if compared with DOHA's headcount method described in Table 2 [a] and we found 100 more GPs each year if compared with DOHA's FTE method described in Table 2 [c].

**Table 2:** Comparing the estimates of the number of general practitioners in the ACT from seven sources with the number of GPs and general practices obtained from the ACT Kindergarten Screen between 2001 and 2008

Year	Methods used to count numbers of GPs in the ACT							
	Headcount [a]	Headcount [b]	Full-Time Equivalent [c]	Full-Time Workload Equivalent [d]	Primary Care Practitioner [e]	FTE Primary Care Practitioner [f]	GPs and Other Medical Practitioners [g]	GPs named in ACT Kindergarten Health Screen
2001	395	387	201	219				283
2002	382	376	196	212				271
2003	386	383	191	203				269
2004	374	370	187	198	398	350		253
2005	375	373	190	200				255
2006	379	381	194	208			391	274
2007	374	373	205	226			408	281
2008	383	383	208	232	371	317	413	289

[a] Source: DOHA [5]. Number of GPs (major specialty at 30 June) who provided at least one MBS service (Non-referred attendance) during the year at a location within the ACT.

[b] Source: DOHA [5]. Number of GPs (major specialty at 30 June) who provided at least one MBS service (Non-referred attendance) during the year with their main practice location within the ACT division of GP.

[c] Source: DOHA [5]. Number of FTE GPs calculated as the proportion of MBS billing at a location in the ACT divided by the average MBS billing of full-time doctors, capped at 1.

[d] Source: DOHA [5]. Number of FEW GPs calculated as the proportion of MBS billing at a location in the ACT divided by the average MBS billing of full-time doctors, not capped at 1, such that an individual GP who bills above average is counted as > 1.

[e] Source AIHW [6]. Number primary care practitioners whose main field of work is clinician (includes those whose main job is not in private rooms, e.g. Acute Care Hospital, Defence, which may not be reflected in Medicare data) Note, these data are based on medical registration rather than MBS claims. AIHW compares these data with column [a]

[f] Source AIHW [6]. Number FTE primary care practitioners whose main field of work is clinician (includes those whose main job is not in private rooms). Note, AIHW compares these data with column [d] http://www.aihw.gov.au/publications/index.cfm/title/10723

[g] Source Medicare Australia [19]. Number of GPs (major specialty at 30 June) providing category 1 services (Professional Attendance) during the 3 months ending 30 June (Q2) who generate > = $1000 in fees for the quarter (Q2) with their main practice location within the ACT division of GP.

In the 2008, 171 parents nominated one of the six Index general practices as their child's practice: two practices had 100% concordance with 22 and 16 parents, one practice had 99% concordance with 75 parents, one practice had 88% concordance with 26 parents, one practice had 77% concordance with 26 parents, and one practice had 67% concordance with 6 parents respectively. The overall mean concordance found matching a child listed in the practice records and the parent having named the practice was 89%, (Chi squared = 24.041, df = 5, p < 0.001).

**Figure 1:**
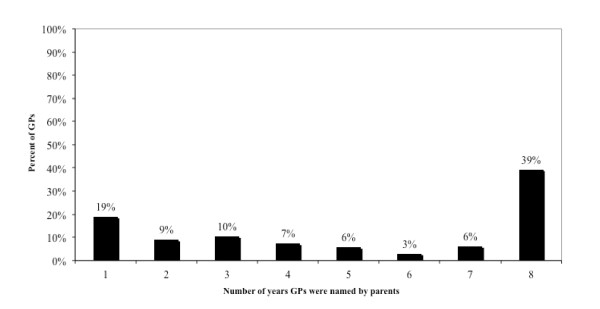
**Percent of GPs named by parents in each year from 2001 to 2008 in the ACT region n = 433 GPs**.

Figure 1 shows the frequency of GPs displaying continuity of care varied over time with 19% of GPs named in only one year and 39% of GPs were named in each of the eight years studied. An average of 324 GPs (92%) were reported by parents to be working in one general practice over the eight years of this study. An average of 26 GPs (7%) were reported to be working in two general practices, and an average of 3 GPs (< 1%) were reported to be working in three general practices in the ACT region.

Table 3 shows that in each year, an average of 22 GPs (8%) were categorised as *new GPs*, an average of 21 GPs (8%) were categorised as *GP left area*, and an average of 13 GPs (5%) were categorised as *displaying transient care*.

**Table 3:** Comparing the total number of GPs with the number of new GPs, GPs who left the area, and GPs displaying discontinuity of care between 2001 and 2008

Year	Total number of GPs	New GP		GP left area		GP displayed discontinuity of care	
		n	%	n	%	n	%
2001	283	NA1*	NA1	NA1	NA1	NA1	NA1
2002	271	28	10%	25	9%	13	5%
2003	269	16	6%	13	5%	14	5%
2004	253	11	4%	20	8%	13	5%
2005	255	15	6%	14	6%	12	5%
2006	274	41	15%	21	8%	16	6%
2007	281	19	7%	21	8%	9	3%
2008	289	22	8%	30	11%	NA2*	NA2

NA1* Means not applicable as a GP can only be counted in this category if their status in 2000 is known.

NA2* Means not applicable as a GP can only be counted in this category if their status in 2008 is known

Table 4 shows the number of general practices worked per GP over the eight-year period. A total of 64 GPs (15%) displayed transient care and fewer parents nominated them as their child's GP compared to GPs who displayed continuity (mean 3.5 versus 11.3 children per GP, t-value = 11.71, df = 2312, p < 0.001). Furthermore, more GPs displayed transient care if parented nominated them as working in large practices compared to small practices (mean 3.3 versus 2.1 GPs per practice, t-value = -3.81, df = 132 p < 0.001).

**Table 4:** The number of general practices worked per GP during each year between 2001 and 2008 in the ACT

Year	Number of Practices per GP						Total
	GP worked in one practice during the year		GP worked in two practices during the year		GP worked in three practices during the year		
	n	%	n	%	n	%	n
2001	319	94%	19	6%	2	1%	340
2002	315	92%	23	7%	3	1%	341
2003	307	91%	27	8%	4	1%	338
2004	307	91%	27	8%	4	1%	338
2005	317	91%	29	8%	4	1%	350
2006	346	92%	29	8%	2	1%	377
2007	356	92%	30	8%	3	1%	389
2008	378	92%	30	7%	3	1%	411

## Discussion

The provision of continuity of care is considered an achievable standard in Australian general practice by the RACGP [4]. To the best of our knowledge, this is the first study to describe how general practitioners achieved this standard by providing continuity of provider care over time. We rejected our first hypothesis to find a greater proportion of GPs displayed continuity of provider care, rather than transient care, but that only 39% of GPs appeared to have been present in the ACT for the full eight years of the study.

Our study also described for the first time how GPs displayed two different forms of transience -either by working in more than one practice in each year or by not being present in the ACT region from one year to the next. From the parents' perspective such GPs ran the risk of impairing longitudinal and personal continuity of care for children.

The long-term lack of GPs within the ACT region has been documented [9]. Our study indicated that GP attrition was an unlikely cause of transient because in each year we found slightly more GPs arrived in the ACT (22), compared to leaving (21). Furthermore, the different methods of counting GPs within the ACT (Table 2) all agreed that the number of GPs remained constant over time. However, the small level of GP turnover would give the appearance of transience. Those parents who were used to seeing only one GP for their care, would perceive transience as a threat to longitudinal continuity of care when forced to see a range of GPs over time. Furthermore, the 15% attrition in the numbers of general practices was due to the loss in the number of solo or small GP practices in the ACT. Our study found small practices were less likely to have GPs who displayed transient provider care. Therefore their loss would add to the parental perception of transient GPs working in the ACT.

Various combinations of GP work patterns will influence how Australians conceive continuity of care. More research is needed to determine whether a GP who works two sessions a week in one practice, or a GP who works two sessions a week in two different practices, is perceived as providing the same kind of continuity of care as the GP who works full time in one practice. Recent surveys of the Australian GP suggest that the future GP is unlikely to want to work in one place, full time, for a lifetime [10]. A 100-year history of continuity of care in New Zealand found that a minority of GPs provided longitudinal continuity of care, as did a minority of general practices (with only 2.8 percent of practices remaining at one address for 30 years or more) [11]. The RACGP standards for general practice unfortunately do not comment on how provider continuity might be achieved in practice [4]. The lack of comment on provider continuity in the RACGP standards impedes academic and policy development on what it means for Australian GPs to provide health care over the long term and how such care shapes our understanding of general practice [12-15].

We rejected our second hypothesis to find disparities in equity of access to GP services by children. There were a constant 12% of children in each year whose parents reported their child did not have a usual medical practitioner. There was a doubling in the proportion of parents (25%) who could not name a GP for their child if they were of Aboriginal or Torres Strait Islander descent or their child was born overseas. This disparity has been found in linguistic studies of provider care in Australia [16]. The observed decline in the number of solo or small practices and the concomitant rise in the number of large practices had no effect on the constant proportion of parents reporting disparities over time. This indicates that changes on the structure of practices did not affect the overall rate of nominating a GP or equity of access to general practices in the ACT. However, it might be conjectured that the small, but constant, proportion of provider transience by GPs may have had a ripple effect on access in the ACT. One study in New Zealand has shown that the size of a general practice was influenced by word of mouth [17,18]. Parents might report on their experience of transient GPs to other parents who might then choose not to nominate that practice for their child. Evidence on such social effects requires qualitative studies of why parents choose a new practice or to leave a practice.

The first limitation in this study was the parental bias in naming GPs and their practices. This limitation was offset by our systematic method of checking names, by the high whole-of-population response rate (89%) achieved each year, and by the distribution of kindergartens matching the spread of general practices across the whole of the ACT region. Secondly, our method was less likely to identify those GPs who work part-time, or work in more than three general practices, or choose not to care for children. DOHA's headcount method indicted that we might have underestimated the counts by 130 GPs each year. However, Medicare data may lead to overestimates of headcounts because an individual GP can have many provider numbers, one for each state and one for each practice where they work [19]. Finally, this study has not described directly the full complexity of equity of access to GP services for children. However, the data points towards a disparity in equity of access suggesting further policy research is needed to identify possible causes and consequences of such inequality.

## Conclusions

The ACT is a wealthy region of Australia and yet it has a low number of GPs compared to other States and Territories [20]. Many GPs provide continuity of care while some display transient care. Children of Aboriginal or Torres Strait Islander descent or children born outside of Australia appear to experience inequality in provider continuity from their GP. This might disappear if a system of voluntary registration of children was adopted in Australian general practice.

## Abbreviations

ACT: Australian Capital Territory; AIHW: Australian Institute of Health and Welfare; DOHA: Department of Health and Aging; FEW: Full-Time Workload Equivalent; FTE: Full-Time Equivalent; GP: General Practitioner; MBS: Medical Benefits Scheme; PracNet: a Primary Health Care research network in the ACT region; RACGP: Royal Australian College of General Practitioners

## Competing interests

The authors declare that they have no competing interests.

## Authors' contributions

GR helped conceive of the study and helped to draft the manuscript. SC contributed to the design of the study and performed the statistical analysis. CS participated in data collection and helped to draft the manuscript. MK conceived of the study, and participated in its design and coordination and helped to draft the manuscript. All authors read and approved the final manuscript.

## Pre-publication history

The pre-publication history for this paper can be accessed here:

http://www.biomedcentral.com/1471-2296/12/106/prepub

## References

[B1] Huygen FJA Family Medicine. The medical life history of families 1978 New York: Brunner/Manzel

[B2] RACGP | History http://www.racgp.org.au/history

[B3] Saultz JW Defining and measuring interpersonal continuity of care Ann Fam Med 2003 1 3 134 143 10.1370/afm.23 15043374 PMC1466595

[B4] Royal Australian College of General Practitioners Standards for general practice 2010 4 Melbourne: The Royal Australian College of General Practitioners 149

[B5] General Practice Statistics http://www.health.gov.au/internet/main/publishing.nsf/Content/General+Practice+Statistics-1

[B6] Medical labour force http://www.aihw.gov.au/publications/index.cfm/title/10723

[B7] Korda RJ Butler JR Clements MS Kunitz SJ Differential impacts of health care in Australia: trend analysis of socioeconomic inequalities in avoidable mortality Int J Epidemiol 2007 36 1 157 165 17213209 10.1093/ije/dyl282

[B8] Phillips CBYR Glasgow NJ Ciszek K Attewell R Improving response rates to primary and supplementary questionnaires by changing response and instruction burden: cluster randomised trial Australian and New Zealand Journal of Public Health 2005 29 5 457 460 10.1111/j.1467-842X.2005.tb00226.x 16255448

[B9] ACT GP Taskforce Kljakovic M General Practice and Sustainable Primary Health Care - The Way Forward 2009 Canberra: ACT Health 41

[B10] About one in ten doctors plan to quit over the next 5 years https://mabel.org.au/Media/23042009-results.htm

[B11] Kljakovic M Continuity of care provided by general practice in Wellington over 100 years New Zealand Family Physician 2008 35 1 16 21

[B12] Dammery D Fledgling general practice in Australia Aust Fam Physician 2001 30 8 808 809 11681158

[B13] Gandevia B A history of general practice in australia Can Fam Physician 1971 17 10 51 61 20468689 PMC2370185

[B14] Leavesley J A history of general practice Med J Aust 1984 141 2 107 109 6377028 10.5694/j.1326-5377.1984.tb132715.x

[B15] Strasser R The origins of general practice Med J Aust 1991 155 9 609 611 1943958

[B16] Ou L Chen J Hillman K Health services utilisation disparities between English speaking and non-English speaking background Australian infants BMC Public Health 2010 10 182 10.1186/1471-2458-10-182 20374663 PMC2858120

[B17] Pilotto LS McCallum J Raymond C McGilchrist C Veale BM Sequential continuity of care by general practitioners: which patients change doctor? Med J Aust 1996 164 8 463 466 8614335 10.5694/j.1326-5377.1996.tb122121.x

[B18] Tracey J Barham P Survey of North Shore residents' views of general practice New Zealand Family Phisician 2001 28 1 1 8

[B19] Statistics http://www.medicareaustralia.gov.au/about/stats/index.jsp

[B20] Australian Government General Practice in Australia: 2004 2005 First Canberra: GP Communications and Business Improvement Unit. Primary Care Division. Department of Health and Aging

